# Informal payments for inpatient health care in post-health transformation plan period: evidence from Iran

**DOI:** 10.1186/s12889-020-8432-3

**Published:** 2020-04-20

**Authors:** Leila Doshmangir, Haniye Sadat Sajadi, Maryam Ghiasipour, Ali Aboutorabi, Vladimir Sergeevich Gordeev

**Affiliations:** 1 Social Determinants of Health Research Center, Tabriz Health Services Management Research Center, Iranian Center of Excellence in Health Management, School of Management and Medical Informatics, Tabriz University of Medical Sciences, Tabriz, Iran; 2 Knowledge Utilization Research Center, University Research and Development Center, Tehran University of Medical Sciences, Tehran, Iran; 3 Department of Health Management and Economics, School of Public Health, Tehran University of Medical Sciences, Tehran, Iran; 4 School of Management and Medical Informatics, Iran University of Medical Sciences, Tehran, Iran; 5 Department of Infectious Disease Epidemiology, The London School of Hygiene & Tropical Medicine, Keppel Street, London, WC1E 7HT UK; 6 Institute of Population Health Sciences, Queen Mary University of London, Mile End Road, London, E1 4NS UK

**Keywords:** Health expenditures, Informal payments, Iran, Health care reform, Health policy, Health policy and systems research

## Abstract

**Background:**

In 2014, a revision of the national medical tariffs for inpatient health care services took place in Iran, and a new hotline was set up to report informal payments. It was expected that such measures would eliminate or decrease informal payments prevalence. This study estimates the prevalence of informal payments for inpatient health care services in the post-reform period, explores factors associated with informal payments and examines patients’ and healthcare providers’ views regarding the causes of informal payments and possible practical solutions for their reduction.

**Methods:**

We surveyed by phone patients who used inpatient health care services in seven Iranian hospitals in 2016. Descriptive and regression analyses were used to estimate the prevalence and determine factors associated with informal payments. We conducted a qualitative analysis through thematic analyses based on focus group discussions and in-depth interviews.

**Results:**

Of 2696 respondents, 14% reported paying informally for inpatient services. Informal payments were reported more frequently among private hospital users, given more frequently to physicians in public teaching hospitals and ‘other staff’ in private hospitals, in the form of cash and voluntary. Being an adult, hospital or treatment type, being insured, and household head’s education influenced the probability of paying informally. The amount paid informally was associated with being insured, the educational status of the household’s head, household size, service, and hospital types. Based on qualitative findings, the leading causes of informal payments reported by patients and healthcare providers can be categorized into four groups - financing challenges; governance challenges; service delivery challenges; and actors and stakeholders. Modifying, adjusting and applying policy interventions; supervision, monitoring and evaluation; and actors and stakeholders were identified as possible solutions for tackling informal payment in the inpatient health care services.

**Conclusion:**

The prevalence of informal patient payments for inpatient services in the post-reform period seems to have reduced; however, they remain to be common. Regular monitoring, reviewing of payment policies to the physicians, informing patients, changing the behaviour of healthcare providers and patients, and developing ethical guidelines to prevent informal payments were suggested for reduction and elimination of informal payments in the Iranian healthcare sector.

## Background

Informal payments (IPs) in healthcare can be defined as a payment made by a patient (or anyone else acting on behalf of this patient) to a provider (person or institution) that is paid in addition and/or in excess to what is officially being determined as a service fee [1, 2]. IPs can be made in cash, given as a gift or by providing a service. A considerable amount of out-of-pocket payments in the health systems of many countries is informal [3, 4]. IPs are one of the significant obstacles in reforming healthcare systems due to their possible adverse effects on equity [5, 6]. IPs can also lead to an undefined financial relationship between a physician (and other medical staff) and a patient, impose a double fiscal burden onto a patient and their family and, in the long run, can damage the community’s trust into health care services providers [7]. Moreover, IPs may increase the catastrophic health expenditure, overshadow the availability and utility of services, decrease the quality of services, increase corruption, decline the confidence and transparency in the system, cause suspicion over the responsibilities of institutions and negatively impact healthcare financing systems [8–11]. Due to possible consequences, reducing the prevalence of IPs for health care services should remain the top priority for health policymakers. The Islamic Republic of Iran (Iran) has a complex healthcare system that includes multiple providers of health care services, financed by public and private sources, and has three levels of health care services provision. For inpatient services, a provider payment mechanism is historically rooted in salaries and the fee-for-service payment model. From 2010 to 2015, the Current Health Expenditure (CHE) per capita decreased from 380 to 366 USD; public expenditure on health as percent of CHE increased from 32 to 53%, and the share of out-of-pocket expenditure from CHE decreased from 59 to 40% [12]. Nonetheless, despite numerous reforms and policy interventions to improve healthcare financing, equitable financing remains to be one of the significant challenges faced by the country’s healthcare system [13]. The Iranian healthcare sector also struggles with inefficient risk pooling arrangements and uncapped fee-for-service charges for both inpatient and outpatient services [14–16].

Another remaining challenge is a high prevalence of IPs that can range from 7 to 10% in a hospital department to 20–48% in the whole hospital [17]. One of the main causes of IPs in Iran is low medical tariffs - tariffs incorrectly estimated and not reflect the actual total costs of healthcare services) [11, 18]. Recognizing the possible negative effects of IPs and their root causes, the Iranian health policymakers attempted to tackle IPs by revising the national medical tariffs. During the last decades, such revision took place several times. However, the revision process was neither comprehensive nor systematic, and, as a result, did not yield into a list of well-defined relative and monetary values for medical procedures and services that would reflect real actual costs [19].

The most recent attempt to tackle IPs was made during the implementation of the latest healthcare sector reform in 2014, known as the Health Transformation Plan (HTP). The HTP reform, developed by the Iranian Ministry of Health and Medical Education (MoHME), included several interventions to boost the quality and equity in the healthcare delivery system. Two elements of the HTP reform addressed IPs: national medical tariff revision and setting up a new hotline “1690” to report IPs. The revision of national medical tariffs was conducted based on feedback from the major healthcare sector stockholders and aimed to reach actual medical costs [20]. All inpatient services that are provided through the Iranian health system were covered under the new national medical tariffs schedule. This new “1690” hotline was created to provide a feedback mechanism and enable patients to file complaints related to an extra payment requested by any medical staff for any medical service in excess of those specified in the medical tariffs schedule. Following a complaint, experts would conduct a speedy investigation to check all cases and related health care providers. Overall, it was expected that the implementation of the newly revised national medical tariffs schedule coupled with a new hotline would eliminate the IPs or at least decrease their prevalence. Whether this happened or not remains to be a very relevant but understudied question for the health policymakers. This study examines the evidence to provide an answer to this question. It is worth noting that the evaluation of the HTP itself as reform is outside the scope of this paper.

The aims of the study were: [1] to estimate the prevalence and factors associated with reported IPs for inpatient health care services in the post-HTP period; and [2] to explore perceptions of patients and healthcare providers regarding the causes of IPs in inpatient settings and practical policy solutions that could be implemented in Iran based on these perceptions.

## Methods

We used a sequential explanatory approach to analyze primary data collected during a national cross-sectional survey, supplemented by a focus group discussion, semi-structured in-depth interviews, and short interviews on the causes of IPs for inpatient services in the period following the revision of the national medical tariffs schedule, as well as possible practical solutions to eliminate them. Both qualitative and quantitative data collection took place in 2016.

### Quantitative phase

To address aim [1], the quantitative data were collected using a questionnaire that was previously tested and used in the Iranian setting [21], which included IPs questions used in other studies as well [7, 22, 23]. Our respondents were patients (or their family members) who were discharged from a hospital within one month prior to the start of the data collection. Respondents were interviewed by phone by a group of trained interviewers using the local language spoken in a corresponding province. The questionnaire included questions on demographics and patient insurance characteristics; the duration of the patient’s stay; experience of giving IPs, i.e. who received the payment (physician or other staff, which included nurses, midwife, security guards, therapists, technicians, clerical staff, and administrative staff), reason for paying IPs (requested by a service provider or done by patient’s initiative), form of payment (cash or in-kind), and the approximate amount of payment (for those who paid in cash).

Participants were selected using a multiple-stage random sampling technique to acquire a country-representative sample from various Iranian hospitals. First, we purposefully selected four large provinces from north, south, west and east of Iran as clusters and considered all existing hospitals there. Second, in each province, we randomly selected two hospitals that served as the main healthcare providers in that province, irrespective of their organization type and ownership category (public teaching, private or affiliated with the social security organization). Hospitals declining to participate in the study were removed from the list and replaced by another randomly selected hospital. Third, using a hospital’s discharge list (last month), respondents were selected using systematic random sampling.

A cluster sample size (*n* = 312) was calculated assuming 5% precision, 95% confidence level, and 28.2% expected IPs proportion (tested by us in a pilot study on 100 respondents). Assuming the intra-cluster correlation coefficient 0.002, the design effect was 1.622. We set expected non-response rate to 30% to account for the sensitive nature of the topic, possible difficulties in accessing the target population, and the highest previously reported non-response rate for telephone interviews on IPs in Iran (27.3%) [24, 25]. The final target sample size was at a minimum of 2888 respondents.

With the Gaal’s definition for IPs in mind [8], all extra payments (whether given in cash or in-kind, voluntary or upon request) over the determined formal tariff schedule paid by the patient (or their family members) were considered as IPs. It is worth noting that since IPs constitute a very sensitive topic and in fact are illegal (following the Iranian legislation and particularly after the revision of the medical tariffs schedule), it was very unlikely that respondents would answer questions regarding IPs freely and honestly. To account for that, we inquired about IPs using the following exact formulation of a question for cash payments: “*Did you or any of your family members make any payments in addition (extra) to the registered formal payment shown in your hospital bill to a physician or other hospital staff?*”. The follow-up questions asked to specify whether these payments were made to a physician, and/or other medical staff, as well as the amount paid. For non-cash in-kind payments, the follow-up question was formulated as follows: “Apart from payment in cash, what kind of other payments did you make?”, giving the following predefined options: flower and gift, goods, or other services. When asking these questions, we did not exclude cases with children’s hospitalization.

The prevalence of IPs was estimated using descriptive statistics. The two-part model was used to analyze factors associated with the likelihood of paying IPs (probit model) and its amount (generalized linear model with gamma distribution and log link function). All quantitative data were analyzed using STATA SE 15.1 (Stata Statistical Software: Release 15. College Station, TX: StataCorp LLC, 2017).

### Qualitative phase

To address aim [2] and to obtain an in-depth understanding of the reasons behind IPs prevalence for inpatient services after revising medical tariffs schedules, the qualitative data were collected by conducting a focus group discussion with 12 participants and 18 face-to-face semi-structured in-depth interviews. Participants’ details can be found in Additional file 1. All participants were purposefully selected among healthcare providers and health policymakers (i.e. physicians, health insurance organizations and MoHME officials, managers and heads of hospitals, heads of financial and administrative affairs in hospitals, faculty members and researchers), according to their position and participation in HTP implementation, as well as their work experience in the inpatient services. Moreover, additional short phone interviews were conducted with a subsample of 364 respondents who reported paying IPs, selected from the primary survey sample.

All face-to-face interviews were carried out until the data saturation point was reached, meaning that there were no new codes identified in the successive interviews. The interviews were conducted using a semi-structured interview guide containing the results of the quantitative part of the study. Participants were asked to answer why the prevalence of IPs is still considerable despite the revision of the medical tariffs schedule; what factors could possibly be influencing this phenomenon; are there any other factors (other than the medical tariffs schedule) that policymakers have not yet addressed; and what practical policy options, intervention and/or solutions can be used to eliminate and/or to prevent this type of payments in the healthcare sector. All interviews and focus group discussions were recorded only after obtaining verbal consent from the respondents. The recorded discussions and interviews were then transcribed verbatim following each session. Also, various documents, including the bylaws, newspapers, rules and regulations, as well as national related reports, were reviewed. All qualitative data were analyzed through inductive (remaining open to accommodate emerging themes) and deductive (framework analysis method) approaches. We applied a framework analysis method and followed five main steps: familiarisation, identifying a thematic framework, indexing, charting and mapping, and interpretation. MAXQDA12 software was used to organize the analysis (VERBI Software. MAXQDA 2018. Berlin: VERBI Software, 2017.).

We also considered the quality criteria, including credibility, transferability, dependability, confirmability and reflexivity for the qualitative research. To ensure credibility, we had a prolonged engagement with all participants to build the necessary level of trust. We also used the triangulation strategy. For transferability, we used a thick description of the context and experiences of participants. Audit trails were used to ensure dependability and confirmability. While conducting qualitative studies, researchers considered the process of critical self-reflection about preferences and preconceptions as researchers to ensure reflexivity.

The ethics approval and consent to participate in this study were obtained. The objectives of the study, data collection methods, data recording, and the role of researchers and interviewees were explained to all participants. Oral informed consent was obtained from all individual participants before inclusion in the study. The data were fully anonymized before the analysis and participants were assured about anonymity.

## Results

### IPs: who is paying and how much

Two thousand six hundred ninety-six patients participated in the study (response rate: 93.3%), with 68.2% from public teaching, 19.5% from private and 12.3% from social security hospitals. The average age of participants and heads of households were 43.2 and 47.6 years, respectively. The mean of household’s income was 28,978,641 IRR per month (ca. 960 USD). Other socio-economic details can be found in Additional file 2.

Overall, 14% of respondents incurred IPs. Of all respondents, 4.1% paid only to physicians, 8.1% paid only to other staff, while 1.8% paid to both doctors and other staff. IPs prevalence was highest in private hospitals (7.2%), followed by public teaching (6.5%) and social security (0.3%) hospitals.

Among those who reported IPs, payments were made more frequently to other staff only (57.9%) than to physicians only (29.4%) or paying to both other staff and a physician (12.7%), with the highest mean amount being paid to the physicians. In public teaching hospitals, IPs were given more frequently to physicians only (57.1%), in private hospitals to other staff only (81.4%), and in social security hospitals also to physicians only (55.6%). Patients also seemed to pay informally more for inpatient services in public hospitals than in private or social security hospitals (Table 1).

**Table 1:** Informal payments for health care services by hospital type

	All respondents			Among those who reported paying IPs					
	Used services in hospital	Given IPs to either physician and/or other staff (*n* = 2696)		Given to a physician only		Given to other staff only*		Given to both a physician and other staff	
Type of hospital	Observed frequency, *n* (%)	Observed frequency, *n* (%)	Amount in USD, mean ± SD	Observed frequency, *n* (%)	Amount in USD, mean ± SD	Observed frequency, *n* (%)	Amount in USD, mean ± SD	Observed frequency, *n* (%)	Amount in USD, mean ± SD
Public teaching	1839 (68.2)	175 (9.5)	164.9 ± 224.9	100 (57.1)	284.9 ± 243.5	57 (32.6)	3.9 ± 18.4	18 (10.3)	94.6 ± 110.6
Private	525 (19.5)	194 (36.9)	30.5 ± 67.1	6 (3.1)	66.3 ± 0	158 (81.4)	12.2 ± 46.3	30 (15.5)	121.8 ± 85.9
Social Security	332 (12.3)	9 (2.7)	6.3 ± 7.9	5 (55.6)	16.6 ± 0	4 (44.4)	1.2 ± 0.6	0 (0.0)	0 ± 0.0
All	2696 (100)	378 (14.0)	90.5 ± 172.1	111 (29.4)	269.7 ± 242.6	219 (57.9)	9.9 ± 40.6	48 (12.7)	111.6 ± 95.7

Source: Authors’ analyses of data from Informal Patient Payments dataset

Notes: * “Other staff” category includes nurses, midwives, security guards, therapists, technicians, clerical staff, and administrative staff. 1 USD = 30,170 IRR in 2016. Reported frequencies are not valid percentages and take all eligible respondents in a group as a denominator, mean values are calculated for valid responses, using the number of service users per type of a hospital as the denominator. The total values of frequencies of payments may vary because of missing answers (lack of response, refusal to respond or respondents did not know the answer). IPs include both cash and/or in-kind contributions, unless stated otherwise

Results regarding forms and incentives for IPs by hospital type and provider should be treated with caution due to a high level of missing responses. Among those who responded to these questions, most IPs were made voluntary and in the form of cash (Table 2). This observation seems to be valid for all types of hospitals and when paid to other staff. For physicians, users reported paying IPs upon request more frequently than paying voluntarily.

**Table 2:** Informal payments for health care services by type of payments

Hospital type		All (user *N* = 2696)		Public teaching (use *N* = 1839)		Private (user *N* = 525)		Social Security (user *N* = 332)	
Who received IP		Physician	Other staff*	Physician	Other staff*	Physician	Other staff*	Physician	Other staff*
Type of payment		Observed frequency, *n* (%)		Observed frequency, n (%)		Observed frequency, *n* (%)		Observed frequency, *n* (%)	
Form	Cash	151 (5.6)	256 (9.5)	114 (6.2)	72 (3.9)	32 (6.1)	180 (34.3)	5 (1.5)	4 (1.2)
	In kind	0 (0.0)	24 (0.9)	0 (0.0)	24 (1.3)	0 (0.0)	0 (0.0)	0 (0.0)	0 (0.0)
	Cash and in-kind	8 (0.3)	11 (0.4)	4 (0.2)	3 (0.2)	4 (0.8)	8 (1.5)	0 (0.0)	0 (0.0)
Incentive	Voluntary	36 (1.3)	265 (9.8)	36 (2.0)	99 (5.4)	*.*ª	162 (30.9)	*.*ª	4 (1.2)
	By request	71 (2.6)	36 (1.3)	37 (2.0)	10 (0.5)	32 (6.1)	26 (5.0)	2 (0.6)	*.*ª

Source: Authors’ analysis of data from Informal Patient Payments dataset

Notes: * “Other staff” category includes nurses, midwives, security guards, therapists, technicians, clerical staff, and administrative staff. 1 USD = 30,170 IRR in 2016. Reported frequencies are not valid percentages and take all eligible respondents in a group as a denominator. The total values of frequencies of payments may vary because of missing answers (lack of response, refusal to respond or respondents did not know the answer).. ª indicates missing answers, where no answers in that category were given

### IPs: factors associated with paying informally and the amount paid

Based on the results of the probit regression analyses (Table 3), the following factors decreased the probability of paying IPs: being an adult (*p* < 0.001) (reference - child), using social security hospital services (=0.001) (reference - public), and residing in a household where household head had a high school education level (*p* < 0.001) (reference – primary level education). Living in a city (*p* = 0.015) (reference - country’s capital, Tehran), being insured (*p* = 0.003), using private hospital (*p* = 0.003) (reference – public hospital), as well as using medical treatment and diagnostic measures or other services (all *p* < 0.001) (reference - surgery) increased the probability of paying IPs. Other factors in the model did not have a significant relationship (*p* > 0.05) with IPs prevalence, or the coefficient values were extremely low.

**Table 3:** Factors associated with IPs prevalence and amount paid (based on Probit and GLM)

Characteristics	Part 1 Paying informally with money, yes/no Yes = 1 (Probit)			Part 2 Amount paid informally GLM	
Variables	Coef (SE)	*p*-value	Marginal effects (SE)	Coef(SE)	p-value
Sex, female	−0.14(0.10)	0.185	−0.01(0.18)	− 0.57 (0.29)	0.052
Adult, yes	−0.88(0.15)	**0.000**	−0.14(0)	1.25 (1.05)	0.236
Residence (ref: country’s capital, Tehran)					
Other city	0.26(0.11)	**0.015**	0.03(0.01)	0.06 (0.29)	0.827
Village	−0.29(0.25)	0.228	−0.03(0.19)	0.22(0.63)	0.722
Insured, yes	0.75(0.25)	**0.003**	0.06(0)	4.76(0.40)	**0.000**
Hospital stay, days	−0(0)	**0.001**	−0(0.86)	− 0(0)	**0.000**
Hospital type (ref: public)					
Private	0.38(0.13)	**0.003**	0.05(0.01)	−1.13(0.48)	**0.018**
Social	−0.7(0.21)	**0.001**	−0.06(0)	−2.78(1.24)	**0.025**
Hospital service (ref: surgery)					
Medical treatment	0.87(0.15)	**0.000**	0.09(0)	1.11(1.02)	0.277
Diagnostic measures	0.80(0.19)	**0.000**	0.08(0)	1.49(1.11)	0.182
Caesarean Section	0.43(0.55)	0.434	0.04(0.5)	−5.290.98)	**0.000**
Other	1.47(0.2)	**0.000**	0.20(0)	0.50(0.97)	0.603
Household size	0.01(0.02)	0.638	0(0.64)	− 0.15(0.05)	**0.002**
Household income, monthly	0(0)	**0.000**	0(0)	0(0)	0.183
Household head, age	−0.03(0)	**0.000**	−0(0)	0(0)	0.735
Household head, an education level (ref: primary)					
High school	−0.60(0.14)	0.000	−0.06(0)	0.92	**0.023**
College	−0.04(0.13)	0.740	−0(0.74)	0.11	0.698
N of respondents	2027			310	
	Prob>chi2 = 0.0000			AIC = 30.10	
	Pseudo R2 = 0.5318			BIC = -120.06	

Source: Authors’ analysis of data from Informal Patient Payments dataset

Notes: Bolding used to reflect *P* values < 0.05. 0(0) values represent extremely low coefficient values

We looked additionally at the probability of paying IPs with cash either to a doctor or other medical staff, or either; the probability of paying informally in-kind to either; as well as the probability of paying informally either in cash or in-kind to either (Additional file 3). Overall, being an adult, living in the city other than the capital, being insured, type of the hospital and treatment, as well as the household head’s education level, all remained to be statistically significantly associated with the probability of paying IPs. There was a slight variation in the direction of the association of IPs predictors when analyzing doctors and other staff separately; however, other than that, the additional regressions only confirmed the main findings.

A positive association of the amount paid informally was found with being insured (*p* < 0.001) and living with a household head who had a high school education level (Table 3). Characteristics such as visiting private and social security hospitals (compared to the public) (*p* = 0.018 and *p* = 0.025 respectively), as well as having a Caesarean section (*p* < 0.001) and living in a bigger household (*p* = 0.002) were all negatively associated with the amount of IPs. Other factors in the model did not have a significant relationship (*p* > 0.05) with IPs amounts, or the coefficient values were extremely low.

### IPs: perceptions of the causes of IPs and ways forward by healthcare providers and users

Participants reported that the frequency of IPs has dramatically decreased following the implementation of the revised medical tariffs schedule. For example, one participant stated, “*Elimination of under the table payments was like a great surgery, if two years ago this issue was mentioned, fortunately, nowadays in a public sector, the amount of under the table [payments] for physicians is nearly zero”* [Senior health policy maker]*.* However, they believed that the full elimination of IPs, as shown by our quantitative analyses, has not yet been achieved and IPs are still present. “*Even after the implementation of HTP, informal payments exist, even though the healthcare system policymakers believed and sometimes said that they could eliminate informal payments, although the amount may be lower than earlier”* [Health Policymaker]*.* According to the interviewees, the leading causes of IPs categorized into four groups: [1] financing challenges, [2] governance challenges, [3] service delivery challenges, and [4] actors and stakeholders.

1. *Financing challenges of the Iranian health system*

Most of the interviewees mentioned that irrational tariffs are the challenge that has affected the Iranian healthcare system for more than thirty years. They declared that the revision of the tariffs schedule that occurred under the HTP could have potentially decreased the prevalence of IPs but would not effectively eliminate it. They also mentioned that after the implementation of the HTP, despite an increase in the amounts paid to medical tariffs, inequity increased among different medical groups. Some of the interviewees believed that increasing medical tariffs as the intervention to control IPs could not be effective without complementary interventions. They reported that despite multiple revisions, tariffs remain irrational, and a revision based on scientific and rational principles of determining health services tariffs is needed. “*In addition to the strict dealing with this phenomenon, the actual price of medical services that would consider interdisciplinary equity should be determined, and insurance organizations should try to increase effective coverage of all people. In this way, we can fundamentally combat under the table payments, but now dealing with this phenomenon is case by case…”* [Health Policy maker]. *“The prominent issue in the healthcare system is the disproportion between the provision of health services and their actual price. Although the medical tariffs underwent considerable change during the implementation of the HTP, as compared to the past revisions, the medical tariffs still do not correspond with economic foundations in the healthcare system”* [Health Insurance Officer]*.*

The payment system, especially for secondary and tertiary healthcare services, which is based on fee for service, is another problem in the healthcare system. Participants believed that the fee for service payment system has caused induced medical interventions, discrimination in physician admission, over-prescription of drugs and resulted in ineffective and expensive services. They mentioned that eliminating a direct relationship between a patient and a provider is a way to move towards the elimination of IPs in the Iranian healthcare sector. *“If we want to minimize the IPs to the level of high-income countries, we should expand public insurance coverage. The health budget should be raised from the public budget. We should not rely on direct payments as a source of health care services financing”* [Health Insurance Officer].

According to some interviewees, if the share of public funding in the CHE does not raise (currently the share of public funding in the CHE is approximately 50% [12]), the country will witness major problems in the healthcare system, including an increased prevalence of IPs. Suitable distribution of resources in the healthcare system based on cost-effective benefit healthcare packages and increasing the efficiency of the health system are possible solutions mentioned by some interviewees.

(2) *Governance challenges of the Iranian health system*

Many interviewees stated that a lack of trust and transparency in the healthcare system and effort to increase one’s revenue is another factor influencing the existence of IPs. Introducing different processes, such as payment mechanisms, supervision and responsibility for transparency and stability in payments are other ways to increase the level of trust in the healthcare system. From their point of view, a lack of required and essential public and private regulations and appropriate laws are very influential on IPs in the country. Preventing a dual practice, when physicians can work in both private and public sector settings, is another solution suggested for IPs elimination. Also, according to many interviewees, obeying transparent legal norms and institutionalization of professional ethics are key factors in the prevention or reduction of IPs: *“Some of the specialists perceive these payments as their right, and they receive these types of payments without feeling any guilt. Surgeons do not usually do the major surgery in public hospitals, and if they do not succeed in receiving under the table payment, they will dispatch the patient to another hospital”* [Patient]. *“Usually, there is no defined and strict mechanism for managing informal payments in private hospitals. Basically, because the heads and stakeholders of the hospitals are physicians themselves, the motivation to deal with under the table payments does not exist. Until now, how many physicians are prohibited from continuing their practice because they received informal payments? None!”* [Faculty Professor]. A fewer number of interviewees said that it is necessary to design and implement programs that can change the attitude and people’s behaviour to prevent appreciation for receiving healthcare through paying IPs. *“This is a tradition, or it is better to say a culture. Our people usually give a gratuity payment or gift to the housekeeping personnel of the hospital. Maybe this is because they think that their income is not proportional to their efforts. Also, housekeeping staff knows this issue too. This tipping is particularly prevalent in a maternity ward. Patients and clients in the obstetric ward usually give tips to the staff without even them asking for it. Sometimes substitution of housekeeping staff in the maternity ward encounters a resistance”* [Senior Staff].

The interviewees reported a lack of supervision and monitoring as a cause of IPs prevalence in the country. MoHME should use its power to stand against the IPs, especially after the HTP implementation, and take serious action to prevent and manage this phenomenon in Iran. *“In my opinion, eliminating/addressing [IPs], especially in the private sector, is not a priority in the healthcare system. I know some specialists that receive tariffs that are more than those approved in their clinics. Some doctors receive the payment for costs from insurance companies and get some money from a patient as well, which is more than the allowed co-payment”* [MoHME’s senior officer]. *“According to the studies, more than 50 percent of outpatient services are provided in private centres, but there is no observable effective combat with IPs in this sector either. In my opinion, the independent institution that is not under the governance of the physicians should have this responsibility”* [Specialist].

From the point of view of many interviewees, it is necessary to monitor the implementation of the revised tariffs schedule continuously. They also referred to a significant role of a “1690” hotline in investigating patients’ complaints about IPs and among suggested punitive measures were: revoking medical licenses, reducing the hospital’s accreditation score, cancelling the insurance reimbursement contracts, dismissal of the heads of public hospitals and cutting bonuses of violators. They also said that by strengthening this role and more serious fight with violators, we could be hopeful to see the decrement in IPs prevalence: *“If someone committed a crime, this person must be identified and charged with it immediately. It is not acceptable that we ignore those who do illegal [activities]”* [Health Researcher].

(3) *Service delivery challenges of the Iranian health system*

Some participants said that despite changes that occurred after implementing HTP, the demand for health services in public hospitals is higher than it was in the past. One of the reasons is that the referral system and a family physician program has not been implemented in full. Increased demand for health care services and failure to extend resources led to longer waiting times and waiting lists for certain services. So, to be able to receive high-quality services and in due time, some patients decide to pay informally. One of the interviewees said: *“Let me give you a real-life example which I recently faced. I work in a heart hospital. The number of patients seeking angioplasty and pacemakers is high, so some must wait several months to receive the service. What happens is that a patient is forced to pay bribery or give a “gift”, or something like that, to a secretary or a receptionist of a doctor, or a physician to reduce their waiting time”* [Hospital Reception Employee]. Most of the interviewees believed that the best solution to eliminate IPs is the implementation of policies such as family physician program and referral system: “*The implementation of family physician program can prevent the bribery and economic offences by cutting a financial relationship between patients and a physician”* [MoHME’s officer].

(4) *Actors and stakeholders*

Many research participants mentioned that the role of actors, such as MoHME and insurance organizations, could potentially have a crucial and vital role in decreasing IPs, as they were involved in many reforms and processes that occurred in the Iranian healthcare system. Furthermore, while health insurance organizations are expected to act as evidence-informed planners and policymakers; in reality, they seem to act only as a channel for redistributing financial resources. Health insurance organizations have a very passive role in determining the quality of the provided services and the ways the payments are made. Nonetheless, most respondents noted that health insurance organizations actually do have a potential and should play an active role in combating unconventional payments: *“The insurance [companies] have a crucial role in facing the phenomenon of IPs, and they can prevent this by defending the rights of the patient and preventing paying extra costs. Strategic purchasing is a strategy that can be useful in decreasing this phenomenon in the health system”* [Iran’s Medical Council officer]. *“I’m sure that if the insurance [company] accomplishes its responsibility to protect a patient, for example by paying the fees to the doctors on time, doctors will not be forced to receive the payments beyond those defined by law”* [Specialist].

Participants also mentioned that health insurance organizations and the way they arrange the reimbursement process to the hospitals and health care providers are very important in dealing with IPs. They think that changes in the health insurance reimbursement level have had a significant effect on inpatient services utilization of public hospitals. One of the participants said: *“The reimbursement rates of public hospitals are now higher than they used to be. But, unfortunately, insurance organizations pay them with some considerable delays. Sometimes these delays can be up to two years. So the hospitals are not able to pay to the providers in time”* [Manager of a hospital]*.*

Some participants believed that IPs are deviating from the norm and represent an abuse of the position. Therefore, the supervision done by organizations including the MoHME, health insurance organizations, the Medical Council and other related organizations is essential as well as co-operation in making a successful effort in tackling IPs. *“The reason for observing IPs in the offices and different districts is the avarice of persons, so these persons must be identified and punished”* [Patient].

Another group of participants proposed that increasing professional responsibility and encouraging patients to use public sector services could be useful to control IPs – something that is already being done in the private sector. *“To be able to tackle IPs, the infrastructure of providing health services in a public sector should be strengthened, the Ministry of Health should use appropriate regulations to eliminate IPs in the country”* [Patient]*.* Some participants mentioned that the perceived discrimination (in monetary terms) in the medical community is another possible reason for asking IPs. One’s perception of being underpaid as compared to peers or even other groups of health care providers validates asking and accepting IPs, which are being seen as well as justified compensation. Participants mentioned that health policymakers and planners should try to set the national medical tariffs more rationally to remove this influential factor.

Informing patients as a main stakeholder of IPs, as well as providers regarding the consequences of asking and giving IPs is another option for reducing the amount of IPs. One of the participants said: *“It is so important to inform patients and give enough information regarding the different types of informal payments. It is necessary to inform that giving different types of gifts is also considered as a voluntary informal payment”* [Policy Advisor].

Some participants mentioned that providers have a critical role in decreasing this phenomenon in the country. They believed that providers should use some strategies to increase the professional responsibility. By professional responsibility, they meant doctors and other staff, improving the quality of care and avoiding getting informal payments in any situation. Some participants also mentioned that MoHME could use the advocacy of other stakeholders, such as the Medical Council to eliminate or prevent this phenomenon in the country. *“All actors and stakeholders should work hand in hand to eliminate this unpleasant phenomenon in the country”* [MoHME officer].

Based on the staff and patients’ views regarding the causes of IPs, related possible practical solutions were grouped into three themes: [1] modifying, adjusting and applying policy interventions [2]; supervision, monitoring and evaluation; and [3] actors and stakeholders (Table 4).

**Table 4:** Themes and subthemes related to causes and practical solutions of IPs

Theme	Factors associated with IPs	Proposed practical solution
Modifying, adjusting and applying policy interventions	Implementing proper reforms and programs	Establishing a referral system and family physician program. Eliminating the direct relationship between a patient and a provider. Using Clinical and Ethical Guidelines. Timely yearly notification of tariffs. Increasing the share of health funding from the public budget.
	Trust to the healthcare system	Increasing transparency in processes in the health system.
	Culture making	Institutionalization of professional ethics. Changing the culture of gratitude through informal payments.
	Mechanisms of tariffs setting	Rationalization of medical tariffs. Setting medical tariffs based on the total cost.
	Payment system	Ratification of financing and payment system. Avoiding any payment inequity between different medical groups.
	Public and private regulations	Preventing the employment of physicians in both public and private sectors (avoiding dual practice).
Supervision, Monitoring and Evaluation	Mechanisms of monitoring and supervision	Organizing systematic supervision, monitoring and evaluation. The proper response to patients’ complaints and following up the demand. The role of MoHME’s in supervision. The supportive role, control and supervision of the Medical Council.
	Mechanisms of reward and punishment	Legislation, preventative regulation conforming with the amount of impact. Strict and efficient dealing with offenders and issuing timely warnings.
Actors and Stakeholders	Role of actors and stakeholders	The supportive role of insurance organizations from physicians and patients. Using the suggestions and advice of all stakeholders. Professional responsibility. Informing patients and other stakeholders.
	Policy advocacy	Strengthening the role of stewardship, regulation and policymaking of MoHME. Avoiding any discrimination in the medical community. Suitable distribution of resources in the healthcare system.
	Health Insurance organizations approach	Avoid reimbursement delays in insurance claims. Effective coverage of people.

## Discussion

IPs remain one of the most challenging issues in Iran’s healthcare system. Policymakers have to address this issue and think about suitable tailored solutions and interventions to be able to control and eliminate it successfully. This is important, as our study confirmed that the prevalence of IPs for inpatient health care services, even after the revision of the medical tariff schedule, is still 14%. These findings correspond to a previously reported range of IPs in the Iranian health care sector of 7–10% in a hospital department to 20–48% in the whole hospital [17], as well as an overall level of IPs in other countries, varying from 2 to 80% [26]. We believe that this relatively low reported prevalence of IPs could be reasonable, given the adjustment of the medical tariffs and the introduction of the new hotline, which allows for real-time feedback by patients who paid IPs and subsequent actions to investigate all cases. Capturing this change was also made possible, as our data was collected 20 months following the HTP implementation.

However, we also acknowledge a possible underestimation of IPs prevalence due to underreporting of such delicate subject (due to its illegal nature following the Iranian legislation), as well as a subjective perception of the patient’s entitlement in regards to existing medical tariffs. For example, despite accounting for a possible 30% non-response rate in sample size calculation, in our study ca. 6.7% of respondents did not give consent to continue with the interview. Also, the majority of our respondents paid IPs voluntary and most frequently to other staff (most likely to thank the underpaid staff for their efforts), predominantly in cash and with the highest prevalence in private hospitals, which can again reflect the lack of knowledge regarding patients’ rights and entitlements. This is despite an extensive media coverage regarding IPs in the health care sector, perhaps, due to an information asymmetry between patients and providers, as patients are not always able to distinguish between what is a formal and what is an informal payment, and what are the exact values of existing medical tariffs and co-payment. As such, these payments always fall in a shadow of ambiguity, particularly when reporting.

Our findings on the IPs prevalence in the post-HTP implementation period seem to be somewhat lower when compared to IPs levels of 21 to 30% reported before the HTP implementation and the revision of the medical tariffs [21, 27, 28]. And even though it was previously shown that prior to HTP implementation the percentage of IPs to physicians in hospitals affiliated to MoHME, social security organization and private sector were 4.5, 8.1, and 12.5% respectively, and no IPs were reported after tariff schedule revision [29]; we do not have sufficient evidence to make a strong conclusion on whether or not the new policies introduced during the HTP implementation helped to control IPs prevalence in the country. This is one of the limitations of the study, given a different sampling frame, data collections techniques, locales and respondents, which makes our findings not directly comparable to those conducted in the pre-HTP period. For future healthcare reforms assessment, we recommend conducting well-planned and timely evaluations, using before and after design, applying similar evaluation methodology, as well as setting up a cohort follow-up, if possible.

We found that the prevalence of IPs was higher in private hospitals, like previous studies conducted in Iran and other countries [17, 22, 26, 29, 30], confirming that the type of hospital ownership is one of the factors contributing to IPs prevalence. We recommend paying more attention when designing future policy interventions to control IPs in the private sector as well. Additionally, we found that while the prevalence of IPs to the physicians was lower than in any other group of providers, physicians are responsible for a substantial proportion of IPs in the country. According to our results, most of the reported IPs were done voluntarily and done to express gratuity and appreciation for receiving high quality and faster services. Fear of receiving a poor quality service and a hope to get a better service was already shown to be among the main reasons for IPs in other studies [6, 30, 31], however, analyzing such reasons was outside the scope of this study.

Our findings indicate that IPs are still prevalent in Iran for inpatient services even after the revision of the medical tariff schedule. The same results were reported in Mekarpour et al. study in 2018. As they showed that IPs are still prevalent in both the outpatient and inpatient services of Iran’s health system and concluded that the HTP has not been entirely successful in eradicating IPs [25]. One probable cause of IPs can be long waiting lists to receive health services from some physicians and a hope to get a better-quality service. Long waiting lists could potentially result from the implementation of some new educational policies in Iran in recent years that led to a limited number of medical students in some major regions of the country with a decreasing specialists’ density in the country with specific profiles [32, 33]. This could explain an increased demand for receiving health care from physicians and finally becoming a motivational factor to request IPs. As such, the monopoly of expertise is another crucial factor of the existing prevalence of some IPs. The integration of medical education into healthcare service stipulated the creation of such a monopoly.

Our findings also showed that IPs are made less frequently for adults than for children. We think that parents are perhaps more willing to pay for their children, and when paying for themselves, they may have more time in circumventing the providers who may demand informal payments, and may use services in which informal payments are less common. The evidence on the effect of age on IPs is mixed [25, 27, 34, 35]. For example, Meskarpour-Amiri et al. reported that the elderly are at increased risk of paying informally for health care, while Zarei et al. found no significant relationship between the IPs behaviour and the patient’s age.

The hospital or treatment type was another factor contributing towards IPs prevalence and amount of IPs, showing that the likelihood of IPs is less for those using social security hospital services in comparison to those who used private hospital services. This finding is similar to other studies that found that the direct financial patient-physician relationship facilitates requesting IPs [11, 36]. The use of medical treatment and diagnostic measures or other services has also increased the likelihood of IPs. It seems that this is related to the ambiguity of medical tariffs for these types of services when compared with surgery services.

We also observed that living in a large city increased the probability of IPs. Living in large cities is usually expensive, while the salary levels of healthcare providers are low. This may cause medical staff to ask for IPs to compensate for the living costs. However, there is no consensus in the literature about the association of the residential area with IPs. Some of them reported that living in the rural areas increased the likelihood of paying IPs [7], while others stated that living in an urban area and large cities increased the probability of paying IPs [37]. In addition, there is some evidence showing no significant relationship between IPs and the place of the patient’s residence [28, 30, 35, 38]. Our study provided evidence to confirm that those living in large cities are at increased risk of paying informally for inpatient health care services.

Our finding also suggests that those residing in a household where the household’s head (who are most likely to be in charge of making any family financial decisions) had high school education seemed less likely to pay informally but more likely to pay higher amounts than others. It implies that highly educated persons were less likely requested to make an IP for inpatient care. However, if they were requested, they would pay more. Perhaps, this reflects only their ability and/or willingness to pay; however, previous studies also provided mixed evidence regarding this relationship [25].

Similar to previous studies, we found that being insured increases the possibility of paying IPs and its amount as well. As Meskarpour et al. explained, it may be due to the fact that having basic health insurance can encourage individuals to receive more services; in such situations, inadequate coverage of cost and services by health insurance can lead to more cost and also IPs by insured individuals [39]. However, some other studies have shown that insured patients are less likely to make IPs [7, 40].

We found that the amount of IPs is less for those patients living in a bigger household than those living in smaller families. This is similar to the findings of previous studies [39, 41, 42]. Most likely, this is because when the family size increases, the ability of a household to pay for health care services costs decreases, due to the household’s budget limitation and intra-household allocations of funds. Also, we think smaller families are more precise regarding seeking a higher quality of care which in turn leads to being more willing to pay informally.

Our qualitative findings that reflected the views of both healthcare providers and health care services users confirmed that even though IPs frequency is not very high after the implementation of the revised medical tariffs schedule, IPs remain and will remain to exist. Among various mentioned reasons were: inactivity of insurance organizations that should act as patients’ rights protectors; inadequate funding and low medical tariffs; necessity to revise payment mechanisms and ensure purchaser-provider split; lack of accountability; lack of punitive measures for those who request IPs and abuse their power and gatekeeper’s function; lack of professional ethics and appropriate professional supervision; lack of continuous supervision, monitoring and evaluation; and delays in reimbursements to the providers. A lack of healthcare system financial resources, low salaries of healthcare providers, low potential of private sector to deliver health services, lack of internal criteria (professional behaviour), delays in timely payments to healthcare providers, and some external criteria (regulations and supervision of their enforcement) were previously reported as the main reasons of requesting IPs by healthcare providers [34, 43, 44].

A complex set of measures is needed to address all these issues in a consistent manner. One of the additional measures could be an introduction of co-payments or of “supplementary fees” on top of the official tariffs set by the health insurance system (e.g. alike those in Belgium and France) [45]. Improvements in the health workforce system and its supply should also be considered. Establishing a referral system based on the strong primary healthcare system, implementing a family physician program, cutting any financial relationship between health care providers and patients are among the main interventions for controlling IPs that should be considered by the Iranian government for future implementation. Strengthening the principles of medical ethics, increasing the level of community awareness in terms of illegal issue of IPs, refining and strengthening the monitoring and supervision mechanisms and developing policies and laws on how to deal with providers who request IPs, as well as increasing awareness of healthcare providers about the negative consequences of IPs, are other effective options in the short term to reduce the IPs. However, these and other possible solutions would require (amongst others) strong government commitment to tackle IPs, substantial revision and optimization of the healthcare system financing model, additional investment in the healthcare sector and appropriate legislative base, as well as substantial information campaigns [46]. As it is evident from a previous study [47], the implementation of official user charges might actually lead to a double burden and co-existence of IPs and official charges.

We used a mixed-method approach that helped us to gain an in-depth understanding and receive detailed information and knowledge about IPs for inpatient services in the country. We aimed at achieving high reliability and credibility through the triangulation of different sources of evidence. The data for this study was collected during the 20 months’ period following the HTP implementation, which could potentially be enough to capture any relevant short-term effects. However, we acknowledge that additional studies are needed to capture long-term effects.

As mentioned earlier, our study did not have a true before and after design, as we used cross-sectional data. However, as mentioned in the Introduction, HTP impact on IPs evaluation was outside the scope of this study and our results are in line with findings from all other studies conducted in pre- and post-HTP periods. Recall bias was another difficulty that we faced, as respondents struggled to recall the exact amount they paid as IPs to physicians. To address this potential bias, we limited the recall period to the nearest discharge period of one month. We also acknowledge that we did not have the information about those participants who were requested to make IPs but did not pay, particularly regarding the amount they were asked to pay. These would constitute the false negatives, particularly in a group of those who reported not paying IPs. We tried to account for it by using the two-part model. We did not collect extensive data on the process of paying informally (a time when it happened, how the amount was negotiated and settled, etc.), as well as other possibly important factors such as patients’ awareness, attitude, and culture toward paying IPs, showing a need for more future more detailed studies. Nonetheless, we were able to acquire a country-representative sample from various Iranian hospitals and presented not only quantitative but qualitative findings as well.

It is worth noting that while providing exact study locations or names of the hospitals that participated in our study could have had some relevance towards a local policymaking process, we have chosen not to do so. Given ethical considerations and existing guidelines and our respect for anonymity, as well as assurances given to participating hospitals, a decision was made not to mention selected hospitals in the publication. Overall, we believe that this type of data could be of use for national or regional benchmarking and decision making, but only given it is being collected systematically and for all hospitals, not just those selected and presented in this or other studies. Such information can be easily misinterpreted outside the context, especially by local and national mass media. Nonetheless, all relevant findings were communicated to participating hospitals for internal use.

## Conclusions

It seems that revising medical tariffs, as well as intensifying supervision measures in the public sector through the introduction of the hotline to report IPs could help to decrease the prevalence of IPs. However, IPs continue to exist with the highest prevalence in the private health sector. Since reducing IPs is essential to ensure achieving Universal Health Coverage, identifying factors that affect IPs and adapting strategies that have worked in other countries to address this issue can help to control or eliminate IPs and promote better health for all citizens. Some of these strategies that could be considered are reforming the health financing, modifying the payment mechanisms, scientific and rational revision of the medical tariffs, developing laws and regulations that would address and bring clarity regarding the IPs, increasing motivation and accountability among health care providers, as well as better designed media campaigns about patients’ rights and medical tariffs. A careful revision of the basic medical package could be another recommendation for health policymakers. Finally, further investigations to measure the extent of IPs in all levels of the health care sector, analysis of multiple factors affecting IPs, as well as examining in detail the process of paying informally are necessary. In this regard, the health policymakers and other actors in the healthcare system could employ the research and training capacity to support continuous monitoring and evaluation of the IPs.

## Supplementary information

**Additional file 1.** Qualitative research respondent characteristics.

**Additional file 2.** Respondents’ socio-economic status characteristics.

**Additional file 3.** Factors associated with informal payments prevalence (based on logistic regression), models comparison.

## Data Availability

The datasets used and/or analyzed during the current study are available from the corresponding author on reasonable request.

## Abbreviations

CHE: Current Health Expenditure
HTP: Health Transformation Plan
IPs: Informal payments
MoHME: Ministry of Health and Medical Education
UHC: Universal Health Coverage
WHO: World Health Organization
