# Correction to: Informal payments for inpatient health care in post-health transformation plan period: evidence from Iran

**DOI:** 10.1186/s12889-020-08924-x

**Published:** 2020-05-25

**Authors:** Leila Doshmangir, Haniye Sadat Sajadi, Maryam Ghiasipour, Ali Aboutorabi, Vladimir Sergeevich Gordeev

**Affiliations:** 1 Social Determinants of Health Research Center, Tabriz Health Services Management Research Center, Iranian Center of Excellence in Health Management, School of Management and Medical Informatics, Tabriz University of Medical Sciences, Tabriz, Iran; 2 Knowledge Utilization Research Center, University Research and Development Center, Tehran University of Medical Sciences, Tehran, Iran; 3 Department of Health Management and Economics, School of Public Health, Tehran University of Medical Sciences, Tehran, Iran; 4 School of Management and Medical Informatics, Iran University of Medical Sciences, Tehran, Iran; 5 Department of Infectious Disease Epidemiology, The London School of Hygiene & Tropical Medicine, Keppel Street, London, WC1E 7HT UK; 6 Institute of Population Health Sciences, Queen Mary University of London, Mile End Road, London, E1 4NS UK

**Correction to: BMC Public Health 20, 539 (2020)**

**https://doi.org/10.1186/s12889-020-8432-3**

The ethics statement in this article [1] incorrectly states that the approval for this study was granted by the ethics committee of IR Iran’s National Institute for Health Research (approved No: 241/M/93195). However, the ethics committee did not exist at the time this proposal was sent to NIHR, and the study was approved by the Research Council of NIHR (Approval No: 241/M/93195″). When this error came to light, the authors sought to obtain retrospective ethical approval from the ethics committee. The NIHR responded that obtaining ethical approval retrospectively is not possible and not necessary, especially, since there was no separate Ethics committee at that time. Furthermore, the study was already approved to be conducted by the Research Council, after taking into account all issues, including ethical considerations. Given the non-invasive nature of the study, the Editor and Publisher have decided to take no further action.
