# Supplementary material for: Informal payments for inpatient health care in post-health transformation plan period: evidence from Iran
Source: BMC Public Health. 2020 Apr 20;20:539. doi: 10.1186/s12889-020-8432-3 (PMC7171751; doi:10.1186/s12889-020-8432-3)
Supplement: Supplementary file 1 — Additional file 1. Qualitative research respondent characteristics. [file 12889_2020_8432_MOESM1_ESM.docx]

# Additional file 1: Qualitative research respondent characteristics

| participants CHARACTERISTICS | N Focus group discussion participants  (N=12) | face to Face semi-structured in-depth interivew  (N= 18) |
| --- | --- | --- |
| Gender |  |  |
| Female | 2 | 4 |
| Male | 10 | 14 |
| Position/level |  |  |
| Health policy maker | 1 | 2 |
| Physician | 1 | 4 |
| Health insurance representative | 2 | 4 |
| MoHME official | 3 | 4 |
| managers and heads of hospitals | 1 | 1 |
| heads of financial and administrative affairs in hospitals | 2 | 1 |
| faculty members and researchers | 2 | 2 |

Source: Authors’ analysis of data from the Informal Patient Payments dataset
