# Supplementary material for: Informal payments for inpatient health care in post-health transformation plan period: evidence from Iran
Source: BMC Public Health. 2020 Apr 20;20:539. doi: 10.1186/s12889-020-8432-3 (PMC7171751; doi:10.1186/s12889-020-8432-3)
Supplement: Supplementary file 2 — Additional file 2. Respondents’ socio-economic status characteristics. [file 12889_2020_8432_MOESM2_ESM.docx]

# Additional file 2: Respondents’ socio-economic status characteristics

| Socio-economic status CHARACTERISTICS | N (valid %) of respondents [N=2696] |
| --- | --- |
| Gender |  |
| Female | 1151 (42.7) |
| Male | 1486 (55.1) |
| Patient’s age | 47.5 ± 19.8 |
| Age categories |  |
| Children < 18 years old | 416 (15.4) |
| Adults ≥ 18 years old | 2237 (83.0) |
| Household Head age, mean (years), ± SD | 47.5 ± 19.8 |
| Household Head’s education level |  |
| Illiterate/low literate | 1518 (56.3) |
| High school | 505 (19.2) |
| College | 600 (22.8) |
| Other | 6 (0.2) |
| Basic health insurance coverage |  |
| Yes | 2582 (96.1) |
| No | 106 (3.9) |
| Place of residence |  |
| Urban | 2484 (92.8) |
| Rural | 193 (7.2) |
| Household Income in Iranian Rial, mean (IRR),± SD, per month | 28978641.9 ± 300928100.4 |
| Household Income in US Dollar, mean (USD),± SD, per month | 960.5 ± 9974.4 |

Source: Authors’ analyses of data from the Informal Patient Payments dataset.

Notes: Percentages are rounded and might not add up to 100 per cent due to missing values. 1 USD = ca. 30170 IRR in 2016. We kept the original values reported by the respondents.
