# Supplementary material for: Informal payments for inpatient health care in post-health transformation plan period: evidence from Iran
Source: BMC Public Health. 2020 Apr 20;20:539. doi: 10.1186/s12889-020-8432-3 (PMC7171751; doi:10.1186/s12889-020-8432-3)
Supplement: Supplementary file 3 — Additional file 3. Factors associated with informal payments prevalence (based on logistic regression), models comparison. [file 12889_2020_8432_MOESM3_ESM.docx]

# Additional file 3: Factors associated with IPs prevalence (based on logistic regression), models comparison

|  | Probability of paying informally with cash, yes/no | | | | | | Probability of paying informally with non-monetary, yes/no | | Probability of paying informally with cash and non-monetary, yes/no | |
| --- | --- | --- | --- | --- | --- | --- | --- | --- | --- | --- |
|  | To doctor | | To other staff | | To either | | To either | | To either | |
| Characteristics | Yes=1 (Probit) | | Yes=1 (Probit) | | Yes=1 (Probit) | | Yes=1 (Probit) | | Yes=1 (Probit) | |
| Variables | Coef (SE) | p-value | Coef (SE) | p-value | Coef (SE) | p-value | Coef (SE) | p-value | Coef (SE) | p-value |
| Sex, female | -0.40 (0.12) | **0.001** | 0.18(0.16) | 0.248 | -0.14(0.1) | 0.185 | 0.37(0.18) | **0.042** | -0.12(0.1) | 0.234 |
| Adult, yes | -0.08 (0.21) | 0.711 | -1.10(0.2) | **0.000** | -0.88(0.15) | **0.000** | -0.66(0.29) | **0.024** | -0.88(0.14) | **0.000** |
| Residence (ref: Tehran) |  |  |  |  |  |  |  |  |  |  |
| Other city | 0.49(0.12) | **0.000** | -0.36(0.17) | **0.034** | 0.26(0.11) | **0.015** | 0.39(0.19) | **0.037** | 0.25(0.1) | **0.015** |
| Village | 0.03(0.28) | 0.900 | -0.20(0.32) | 0.524 | -0.30(0.25) | 0.228 | 0.28(0.37) | 0.457 | -0.34(0.24) | 0.152 |
| Insured, yes | 0.72(0.36) | **0.044** | 0.79(0.32) | **0.013** | 0.75(0.25) | **0.003** | -0.10(0.34) | 0.772 | 0.75(0.25) | **0.002** |
| Hospital stay, days | -0.01(0.01) | 0.150 | 0(0) | 0.857 | 0(0) | 0.859 | 0(0) | 0.801 | 0(0) | 0.949 |
| Hospital type (ref: public) |  |  |  |  |  |  |  |  |  |  |
| Private | -0.44(0.15) | **0.003** | 1.16(0.18) | **0.000** | 0.38(0.13) | **0.003** | -0.01(0.22) | 0.958 | 0.33(0.12) | **0.007** |
| Social | -1.02(0.24) | **0.000** | 0.32(0.35) | 0.368 | -0.70(0.21) | 0.859 | -0.54(0.43) | 0.208 | -0.71(0.2) | **0.000** |
| Hospital service (ref: surgery) |  |  |  |  |  |  |  |  |  |  |
| Medical treatment | 1.00(0.21) | **0.000** | 0.35(0.2) | 0.077 | 0.87(0.15) | **0.000** | 0.39(0.26) | 0.131 | 0.84(0.14) | **0.000** |
| Diagnostic measures | 0.76(0.25) | **0.002** | 0.53(0.28) | 0.058 | 0.80(0.19) | **0.000** | 0.74(0.3) | **0.013** | 0.85(0.18) | **0.000** |
| Caesarean Section | 0(0) |  | 0.94(0.61) | 0.125 | 0.43(0.55) | 0.434 | 1.58(0.7) | **0.024** | 0.44(0.55) | 0.427 |
| Other | 0.91(0.25) |  | 1.67(0.26) | **0.000** | 1.47(0.2) | **0.000** | 0.04(0.34) | 0.897 | 1.46(0.19) | **0.000** |
| Household size | -0.08(0.04) | **0.028** | 0.05(0.03) | 0.062 | 0.01(0.02) | 0.638 | 0.03(0.04) | 0.427 | 0.01(0.02) | 0.546 |
| Household income, monthly | 0(0) | **0.000** | 0(0) | **0.038** | 0(0) | **0.000** | 0(0) | 0.164 | 0(0) | **0.000** |
| Household head, age | -0.01(0) | **0.005** | -0.04(0.01) | **0.000** | -0.03(0) | **0.000** | 0(0.01) | 0.787 | -0.02(0) | **0.000** |
| Household head, an education level (ref: primary) |  |  |  |  |  |  |  |  |  |  |
| High school | -0.45(0.16) | **0.005** | -0.27(0.19) | 0.157 | -0.60(0.14) | **0.000** | -0.45(0.29) | 0.115 | -0.53(0.13) | **0.000** |
| College | 0.05(0.13) | 0.673 | -0.07(0.2) | 0.708 | -0.04(0.13) | 0.740 | 0.04(0.21) | 0.852 | -0.05(0.12) | 0.687 |
| N of respondents | 2020 |  | 2027 |  | 2027 |  | 2027 |  | 2027 |  |
| Prob>chi^2^ | 0.0000 |  | 0.0000 |  | 0.0000 |  | 0.0002 |  | 0.0000 |  |
| Presudo R^2^ | 0.2441 |  | 0.7780 |  | 0.5618 |  | 0.1643 |  | 0.5321 |  |

Source: Authors’ analysis of data from Informal Patient Payments dataset

Notes: Bolding used to reflect P values <0.005. Bolding used to reflect P values <0.05.
